# The association of famine exposure with healthy lifestyles at different life stages in rural older adults in China

**DOI:** 10.3389/fpubh.2025.1533909

**Published:** 2025-03-21

**Authors:** Yuxin Sun, Yudong Miao, Saiyi Wang, Yifei Feng, Baoyong Hua

**Affiliations:** College of Public Health, Zhengzhou University, Zhengzhou, China

**Keywords:** famine exposure, healthy lifestyles, healthy lifestyle scores, age balance control, rural older adults

## Abstract

**Objective:**

To assess the potential association between exposure to Chinese famine and healthy lifestyles, as well as any gender disparities in this relationship to provide a scientific basis for the development of effective public health policies and interventions.

**Methods:**

We used binary logistic regression models to estimate the potential association between famine exposure and healthy lifestyles and stratified by sex for comparisons. Unordered multicategory logistic regression model was used to assess the association between famine exposure and healthy lifestyle scores. We presented this association with Odds ratios (OR) with 95% confidence intervals (CI).

**Results:**

A total of 6,458 individuals were enrolled in the current study, of whom 4,155(64.3%) were women. Men exposed to famine in infancy (OR = 0.650, 95%CI: 0.506–0.834) and preschool (OR = 0.788, 95%CI: 0.631–0.985) was negatively associated with non-smoking and women exposed famine in infancy (OR = 0.699, 95%CI: 0.574–0.851), preschool (OR = 0.734, 95%CI: 0.613–0.880), and school age (OR = 0.764, 95%CI: 0.673–0.916) was negatively associated with normal weight, and women were more likely to be central obesity during exposed to famine in infancy (OR = 0.763, 95%CI: 0.624–0.934). The likelihood of having a healthy lifestyle score of 5–6 as an adult was higher for experiencing famine in preschool and school age.

**Conclusions:**

Men exposed to the Chinese famine during infancy were positively associated with smoking and drinking, while women exhibited a positive association with central obesity. Women had unhealthy weight if they experienced famine early in life and a call for attention to nutritional status and women's health in the early life.

## 1 Introduction

In recent decades, the increase in chronic diseases has posed complex challenges to global health care, with diseases such as obesity, cardiovascular disease, and type 2 diabetes closely linked to lifestyle behaviors (1). It has been shown that persistent unhealthy lifestyles or adverse environmental exposures will exacerbate the health effects of famine exposure early in life, leading to increased incidence of chronic diseases such as type 2 diabetes and hypertension (2, 3). Smoking, alcohol consumption, sleep deprivation, physical inactivity, and poor dietary choices are key modifiable risk factors for chronic diseases. Studies have shown that the incidence and severity of chronic diseases can be significantly reduced through smoking cessation, alcohol cessation, healthier physical activity, adequate sleep, and a healthy diet (4, 5).

In the late 1950s and early 1960s, China experienced an unprecedented famine, which resulted in malnutrition and deteriorating health for a large number of people (6, 7). This historical event provided a unique opportunity to gain insight into the long-term effects of malnutrition over a relatively long period of time. In recent years, several studies (8–10) have shown that poor nutritional environments in early life may have profound effects on the long-term health of individuals. Domestic study has found that famine experiences in the early stages of life made individuals experience the hardship of the living environment and the preciousness of material resources, which in turn affects their lifestyles and behaviors in the later stages of their lives (11). Famine experience made individuals realize the importance of material security, formed more conservative behavioral characteristics, and engaged in less social recreation, resulting in the accumulation of stress, depression and other negative emotions, thus affecting mental and behavioral health (12). While there is a large body of research that has explored the impact of early famine experiences on adult health, there is still a need for further exploration of the specific associations with healthy lifestyles. This study focused on the experience of famine during this historical period and analyzed its specific impact on the risk of healthy lifestyle choices of individuals in adulthood.

Unhealthy lifestyles, such as smoking, alcohol consumption, physical inactivity, and obesity, are important risk factors for many non-communicable diseases and may be intermediate between famine exposure and the development of chronic diseases later in life (13, 14). The purpose of this study was to examine the importance of the association between the experience of famine early in life and healthy lifestyles. By exploring this association, the long-term impact of early malnutrition on health status in adulthood can be better understood, thus providing a scientific basis for the development of effective public health policies and interventions to improve overall health.

## 2 Methods

### 2.1 Study participants

In this study, a baseline survey was conducted in Jia County, Henan Province, China, from July 1 to August 31, 2023, and a whole cluster sampling method was adopted in 15 villages and towns in Jia County for patients with diagnosed type 2 diabetes aged 65 years or older who were enrolled in the National Basic Public Health Service Program as the study population. Participants were excluded if they suffered from mental illness or other illnesses that limited their ability to act and were unable to complete the questionnaire and physical examination independently, and we also excluded participants with incomplete information on their date of birth and healthy lifestyle factors (Supplementary Figure S1). We openly recruited interviewers at Zhengzhou University, who were trained in sampling methods, research tools, and quality control, and their skills were subsequently assessed according to a pre-established training program. The study was approved by the Life Sciences Ethics Review Committee of Zhengzhou University (no. 2023-318). All participants were fully informed of the purpose and precautions of the study and signed an informed consent form before the investigation.

### 2.2 Definition of famine exposure

Referring to previous studies on the Chinese famine (15, 16), we determined famine exposure based on date of birth. We divided participants into 4 groups: infancy exposure (born between January 1, 1956, and December 31, 1958, and 0 to 3 years old at the time of the famine), preschool exposure (born between January 1, 1953, and December 31, 1955, and 4 to 6 years old at the time of the famine), school-age exposure (born between January 1, 1950, and December 31, 1952, and 7–9 years old at the time of the famine) and adolescent/adult exposure (born before December 31, 1949, and after age 10 at the time of the famine).

### 2.3 Assessment of health lifestyle factors

In this study, six modifiable lifestyle factors were assessed evaluate healthy lifestyles: smoking, alcohol consumption, physical activity, sleep duration, body mass index (BMI), and waist circumference (WC). Specifically, with regard to smoking, participants were classified as current smokers, former smokers, and never smokers. Current smokers were queried regarding the frequency and quantity of smoking, while former smokers were asked about the age at which they ceased smoking. Never smokers were considered to represent a healthy lifestyle factor (17). In the domain of alcohol consumption, the frequency and quantity of alcohol intake over the past 12 months were recorded. Moreover, former drinkers were asked about the age at which they ceased drinking. Non-drinking was considered a healthy lifestyle factor (18). For physical activity, the frequency and duration of weekly physical activity were documented. A minimum of 150 min of moderate-intensity exercise or 75 min of vigorous-intensity exercise per week was considered a healthy lifestyle factor (19). For sleep, participants were queried about their typical sleep duration and the total number of hours spent asleep per day. A sleep duration of 6 to 8 h was considered a healthy lifestyle factor (20). In terms of body weight and central obesity, the participants' weight and height were measured twice according to the standard protocol, and the mean value was calculated. The BMI (kg/m^2^) was calculated as weight (kg) divided by the square of height (m). Central obesity was defined as a waist circumference of ≥90 cm for men and ≥85 cm for women. Ultimately, these factors were considered healthy when participants had a body mass index (BMI) between 18.5 and 23.9 kg/m^2^ and a waist circumference of <90 cm for men and 85 cm for women [(21); Supplementary Table S1].

All lifestyle variables were defined as dichotomous variables, with a value of 1 assigned for meeting the definition of a healthy lifestyle and a value of 0 for not meeting it. In addition, we categorized each lifestyle factor into healthy and unhealthy levels and assigned a score of 1 and 0, respectively. The healthy lifestyle score was defined as the unweighted sum of the number of healthy lifestyle factors ranging from 0 to 6, with higher value indicating a healthier lifestyle, and was categorized into three groups (0–2, 3–4, and 5–6 scores) by percentile (<P25, P25~, P75~).

### 2.4 Assessment of covariates

The demographic information, solitary status and self-reported diseases history were collected during face-to-face interviews by trained interviewers. Marital status was categorized as married or other (unmarried, divorced, widowed). Education level was classified into illiteracy, primary school, middle school, high school and above. Occupation was categorized as agricultural work or non-agricultural work. Annual household income was calculated by the interquartile range ( ≤ 2,840 RMB, 2,841–5,000 RMB, 4,001–10,200 RMB, and ≥10,201 RMB). In addition, primary health care workers measured the blood pressure (BP) levels of participants three times using a standardized electronic sphygmomanometer, and 1 min among measurements at least. The measurement error did not exceed 10 mmHg, and the mean of the three measurements was used to define the systolic and diastolic BP levels. Self-reported diseases history included hypertension, coronary heart disease and dyslipidemia.

### 2.5 Statistical analysis

We presented categorical variables using frequencies (%), and represented non-normally distributed continuous variables using interquartile range *P*50 (*P*25, *P*75). The Chi-square test or Kruskal-Wallis H test was used to determine whether there were statistical differences among groups, and binary logistic regression models were used to assess the association between famine exposure and a single healthy lifestyle. Model 1 did not include adjustment for confounders, whereas Model 2 controlled for marital status, education, occupation, annual household income, and solitary status. Model 3 added systolic and diastolic blood pressure as well as coronary heart disease and dyslipidemia to model 2. In addition, we stratified by gender to examine differences between men and women experiencing famine exposure and healthy lifestyles.

We used linear regression to further test for the existence of multicollinearity between the independent variables through the results of tolerance and variance inflation factor, if the tolerance is <0.1 or the variance inflation factor is >10, it means that covariance exists. In this study, the tolerance was much >0.1 and the variance inflation factor was <10, so there was no multicollinearity between the independent variables. In addition, we tested the hypothesis of “proportional dominance,” and the results showed that *p* < 0.001, the hypothesis of parallel lines could not be satisfied, so we used unordered multicategory logistic regression to explore the association of the period of exposure to famine on the healthy lifestyle scores, and we used the group of healthy lifestyle scores from 0 to 2 as the reference group. The model was adjusted for sociodemographic factors, major disease history, and blood pressure values.

All statistical analyses were done using SPSS version 25.0, and statistical significance was set at a two-tailed *P* < 0.05.

## 3 Results

### 3.1 Characteristics of study participants

Table 1 showed the baseline characteristics of participants grouped by period of famine exposure. Overall, of the 6,458 adults, of whom 4,155 (63.3%) were female, 1,215 (18.81%) were in the infancy exposure group, 1,487 (23.02%) in the preschool exposure group, 1,326 (20.53%) in the school-age exposure group, and 2,430 (37.63%) in the adolescence/adulthood exposure group. Univariate analysis showed that age, marital status, annual family income, education, occupation, living alone, smoking, drinking, sleep duration, physical activity, weight, waist circumference, major history diseases (coronary heart disease, dyslipidemia), systolic blood pressure, and diastolic blood pressure significantly differed between the famine-exposed groups at different periods (*p* < 0.05).

**Table 1:** Characteristics of study participants.

**Variables**	**Total (*n* = 6,458)**	**Infancy-exposed (*n* = 1,215)**	**Preschool-exposed (*n* = 1,487)**	**School age-exposed (*n* = 1,326)**	**Adolescence/adulthood exposed (*n* = 2,430)**	***P*-value**
**Age in survey**	72.0 (68.0, 76.0)	66.0 (65.0, 67.0)	69.0 (68.0, 70.0)	72.0 (71.0, 73.0)	77.0 (75.0, 80.0)	< 0.001
**Gender, n (%)**						0.682
Male	2,303 (35.7)	422 (34.7)	528 (35.5)	465 (35.1)	888 (36.5)	
Female	4,155 (64.3)	793 (65.3)	959 (64.5)	861 (64.9)	257 (63.5)	
**Marital status, n (%)**						< 0.001
Married	4,817 (74.6)	1,024 (84.3)	1,222 (82.2)	1,021 (77.0)	1,550 (63.8)	
Other	1,641 (25.4)	191 (15.7)	265 (17.8)	305 (23.0)	880 (36.2)	
**Annual household income in CNY/years**, ***n*** **(%)**						< 0.001
≤ 2,840	1,616 (25.0)	215 (17.7)	329 (22.1)	319 (24.1)	753 (31.0)	
2,841–5,000	2,127 (32.9)	386 (31.8)	522 (35.1)	447 (33.7)	772 (31.8)	
5,001–10,200	1,101 (17.1)	248 (20.4)	286 (19.2)	251 (18.9)	316 (13.0)	
≥10,201	1,614 (25.0)	366 (30.1)	350 (23.5)	309 (23.3)	589 (24.2)	
**Education level, n (%)**						< 0.001
Illiteracy	2,885 (44.7)	426 (35.0)	672 (45.2)	684 (51.6)	1,103 (45.4)	
Primary school	2,078 (32.2)	321 (26.4)	423 (28.5)	430 (32.4)	904 (37.2)	
Middle school	1,073 (16.6)	307 (25.3)	283 (19.0)	156 (11.8)	327 (13.4)	
High school or above	422 (6.5)	161 (13.3)	109 (7.3)	56 (4.2)	96 (4.0)	
**Occupation, n (%)**						< 0.001
Agricultural work	4,047 (62.7)	838 (69.0)	998 (67.1)	840 (63.3)	1,371 (56.4)	
Nonagricultural work	2,411 (37.3)	377 (31.0)	489 (32.9)	486 (36.7)	1,059 (43.6)	
**Live alone**						< 0.001
Yes	1,079 (16.7)	132 (10.9)	186 (12.5)	203 (15.3)	558 (23.0)	
No	5,379 (83.3)	1,083 (89.1)	1,301 (87.5)	1,123 (84.7)	1,872 (77.0)	
**Non-smoking**						0.048
Yes	5,145 (79.7)	940 (77.4)	1,173 (78.9)	1,060 (79.9)	1,972 (81.2)	
No	1,313 (20.3)	275 (22.6)	314 (21.1)	266 (20.1)	458 (18.8)	
**Non-drinking**						< 0.001
Yes	5,743 (88.9)	1,022 (84.1)	1,329 (89.4)	1,191 (89.8)	2,201 (90.6)	
No	715 (11.1)	193 (15.9)	158 (10.6)	135 (10.2)	229 (9.4)	
**Healthy sleeping**						0.006
Yes	3,995 (61.9)	794 (65.3)	942 (63.3)	803 (60.6)	1,456 (59.9)	
No	2,463 (38.1)	421 (34.7)	545 (36.7)	523 (39.4)	974 (40.1)	
**Healthy physical activity**						< 0.001
Yes	4,446 (68.8)	934 (76.9)	1,105 (74.3)	961 (72.5)	1,446 (59.5)	
No	2,012 (31.2)	281 (23.1)	382 (25.7)	365 (27.5)	984 (40.5)	
**Normal weight**						< 0.001
Yes	2,426 (37.6)	411 (33.8)	525 (35.3)	482 (36.3)	1,008 (41.5)	
No	4,032 (62.4)	804 (66.2)	962 (64.7)	844 (63.7)	1,422 (58.5)	
**Non-central obesity**						0.018
Yes	2,325 (36.0)	403 (33.2)	537 (36.1)	459 (34.6)	926 (38.1)	
No	4,133 (64.0)	812 (66.8)	950 (63.9)	867 (65.4)	1,504 (61.9)	
**Healthy lifestyle score**						0.017
0–2	943 (14.6)	185 (15.2)	205 (13.8)	166 (12.5)	387 (15.9)	
3–4	3,900 (60.4)	748 (61.6)	884 (59.4)	841 (63.4)	1,427 (58.7)	
5–6	1,615 (25.0)	282 (23.2)	398 (26.8)	319 (24.1)	616 (25.4)	
**Major history diseases, n (%)**						
Hypertension	3,282 (50.8)	588 (48.4)	748 (50.3)	672 (50.7)	1,274 (52.4)	0.136
Coronary heart disease	1,073 (16.6)	174 (14.3)	210 (14.1)	242 (18.3)	447 (18.4)	< 0.001
Dyslipidemia	473 (7.3)	110 (9.1)	128 (8.6)	104 (7.8)	131 (5.4)	< 0.001
**SBP, mmHg**	137.5 (125.3, 150.3)	135.7 (124.0, 147.7)	136.0 (123.8, 148.7)	137.7 (126.0, 151.0)	139.5 (127.0, 152.0)	< 0.001
**DBP, mmHg**	75.3 (68.3, 82.3)	76.3 (70.0, 83.3)	75.5 (69.2, 82.7)	75.7 (68.7, 82.0)	74.3 (66.7, 81.7)	< 0.001

SBP, systolic blood pressure; DBP, diastolic blood pressure.

In addition, the characteristics of the healthy lifestyle scores of the study participants were shown in Supplementary Table S2. Participants with high healthy lifestyle scores were more likely to be female and to have been exposed to famine during adolescence/adulthood. Differences in healthy lifestyle scores were statistically significant (*p* < 0.05) with respect to age, gender, marital status, education, occupation, living alone status, history of major diseases (hypertension, coronary heart disease, dyslipidemia), SBP, and DBP.

### 3.2 Association between exposure to the Chinese famine and healthy lifestyles

As displayed in Table 2, compared to the adolescence/adulthood exposure group, exposure to famine in infancy was positively associated with non-smoking and healthy sleep duration (*p* < 0.05), and the OR and 95% CI of the correlation between infancy preschool, and school-age exposure groups and positive physical activity were 2.324 (1.975, 2.736), 2.022 (1.747, 2.341) and 1.845 (1.591, 2.138). In addition, we found that experiencing famine in infancy, preschool, and school age was negatively associated with normal weight (*p* < 0.05). Experiencing famine during infancy was more likely to develop central obesity in adulthood with an association effect OR (95% CI) of 0.792 (0.679, 0.924; Model 3).

**Table 2:** Logistic regression analysis of famine exposure-related lifestyles.

**Variables**	**Model 1**		**Model 2**		**Model 3**	
	**OR (95% CI)**	***P*-value**	**OR (95% CI)**	***P*-value**	**OR (95% CI)**	***P*-value**
**Non-smoking**						
Adolescence/adulthood-exposed	1.000		1.000		1.000	
Infancy-exposed	0.794 (0.671, 0.940)	0.007	1.121 (1.016, 1.467)	0.033	1.322 (1.097, 1.594)	0.003
Preschool-exposed	0.868 (0.739, 1.019)	0.083	1.058 (0.892, 1.256)	0.516	1.134 (0, 953, 1.350)	0.156
School-exposed	0.926 (0.782, 1.095)	0.368	0.917 (0.768, 1.094)	0.334	0.952 (0, 797, 1.138)	0.591
**Non-drinking**						
Adolescence/adulthood-exposed	1.000		1.000		1.000	
Infancy-exposed	0.551 (0.449, 0.677)	< 0.001	0.840 (0.674, 1.046)	0.119	0.915 (0.732, 1.144)	0.473
Preschool-exposed	0.875 (0.707, 1.084)	0.222	1.085 (0.868, 1.357)	0.473	1.166 (0.930, 1.462)	0.484
School-exposed	0.918 (0.734, 1.148)	0.454	0.923 (0.732, 1.162)	0.494	0.976 (0.733, 1.232)	0.840
**Healthy sleep duration**						
Adolescence/adulthood-exposed	1.000		1.000		1.000	
Infancy-exposed	1.262 (1.093, 1.456)	0.001	1.163 (1.003, 1.348)	0.046	1.173 (1.009, 1.363)	0.038
Preschool-exposed	1.156 (1.012, 1.321)	0.032	1.122 (0.979, 1.285)	0.097	1.122 (0.977, 1.287)	0.102
School-exposed	1.027 (0.896, 1.178)	0.702	1.023 (0.891, 1.175)	0.750	1.036 (0.901, 1.191)	0.618
**Healthy physical activity**						
Adolescence/adulthood-exposed	1.000		1.000		1.000	
Infancy-exposed	2.262 (1.935, 2.644)	< 0.001	2.301 (1.959, 2.702)	< 0.001	2.324 (1.975, 2.736)	< 0.001
Preschool-exposed	1.968 (1.708, 2.268)	< 0.001	2.020 (1.748, 2.333)	< 0.001	2.022 (1.747, 2.341)	< 0.001
School-exposed	1.792 (1.550, 2.072)	< 0.001	1.828 (1.579, 2.117)	< 0.001	1.845 (1.591, 2.138)	< 0.001
**Normal weight**						
Adolescence/adulthood-exposed	1.000		1.000		1.000	
Infancy-exposed	0.721 (0.625, 0.833)	< 0.001	0.726 (0.626, 0.841)	< 0.001	0.782 (0.670, 0.912)	0.002
Preschool-exposed	0.770 (0.674, 0.880)	< 0.001	0.780 (0.681, 0.894)	< 0.001	0.826 (0.717, 0.952)	0.008
School-exposed	0.806 (0.702.0.925)	0.002	0.819 (0.712, 0.941)	0.005	0.855 (0.741, 0.988)	0.033
**Non-central obesity**						
Adolescence/adulthood-exposed	1.000		1.000		1.000	
Infancy-exposed	0.806 (0.697, 0.932)	0.004	0.777 (0.669, 0.902)	0.001	0.792 (0.679, 0.924)	0.003
Preschool-exposed	0.918 (0.803, 1.050)	0.211	0.911 (0.795, 1.044)	0.182	0.924 (0.803, 1.063)	0.267
School-exposed	0.860 (0.748, 0.989)	0.034	0.873 (0.758, 1.005)	0.058	0.888 (0.769, 1.025)	0.104

Model 1 did not adjust for confounders; Model 2 adjusted for marital status, education, occupation, annual household income, living alone status; Model 3 further adjusted for coronary artery disease, dyslipidemia, systolic and diastolic blood pressure based on model 2.

**Figure 1:**
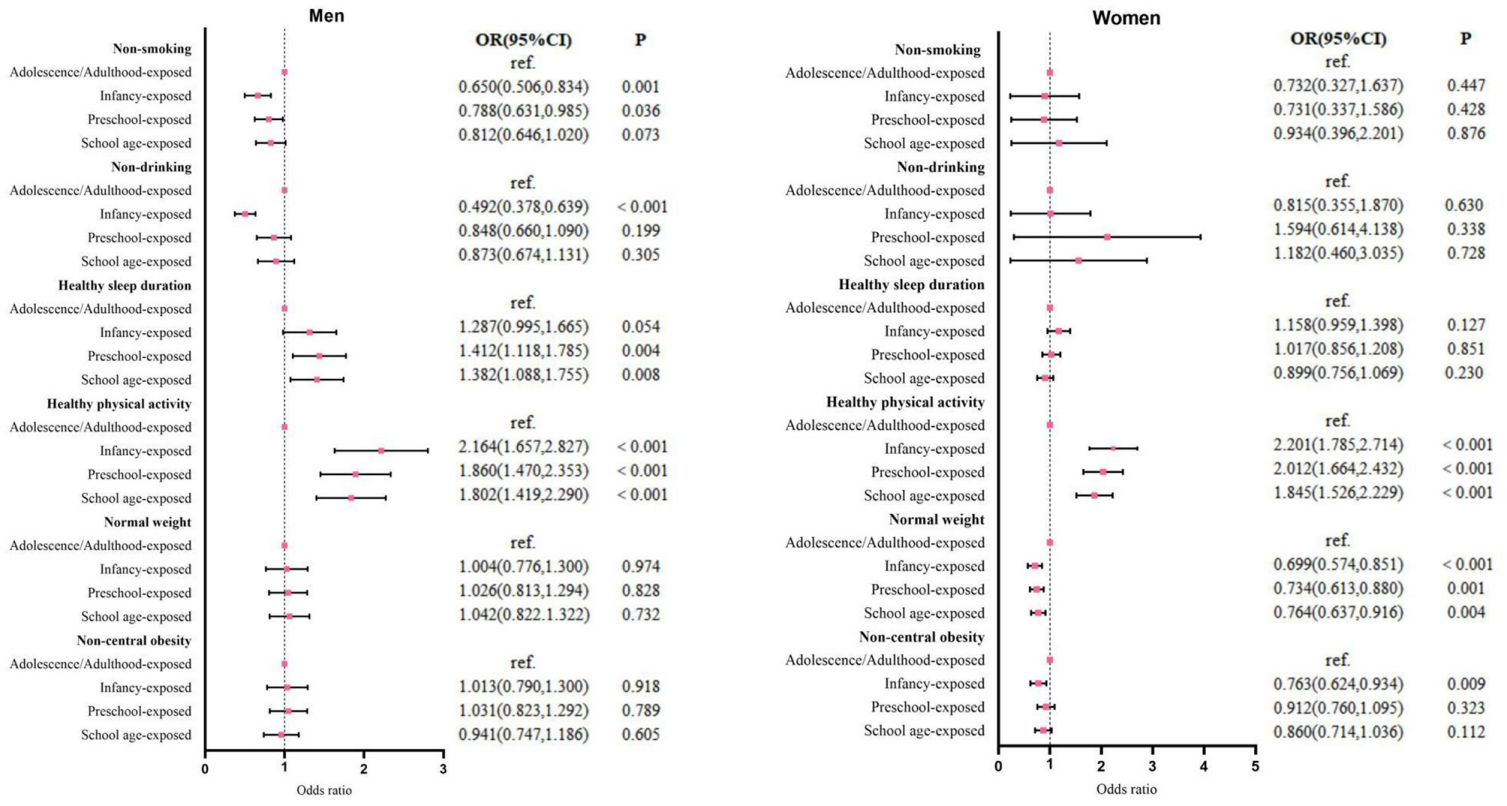
Forest map of the multivariate logistic regression analysis by sex. All analyses control for marital status, education, occupation, annual household income, living alone status, coronary artery disease, dyslipidemia, systolic and diastolic blood pressure.

To further understand how the association between famine exposure and healthy lifestyles differed between men and women, we performed regression analyses stratified by gender. As shown in Figure 1, we found that experiencing famine in infancy (OR = 0.650, 95%CI: 0.506–0.834) and preschool (OR = 0.788, 95%CI: 0.631–0.985) was negatively associated with non-smoking in men, and exposure to famine in infancy was negatively associated with non-drinking and positively associated with healthy sleep duration (*p* < 0.05). We did not find these associations in women. Furthermore, Women famine experience in infancy (OR = 0.699, 95%CI: 0.574–0.851), preschool (OR = 0.734, 95%CI: 0.613–0.880), and school age (OR = 0.764, 95%CI: 0.673–0.916) was negatively associated with normal weight, and women exposed to famine in infancy (OR = 0.763, 95%CI: 0.624–0.934) were more likely to be central obesity in adulthood, with no similar associations found in men. In addition, a strong positive association between famine exposure and healthy physical activity was found in results stratified for both sexes.

### 3.3 Association of famine exposure with healthy lifestyle scores

The association between famine exposure and healthy lifestyle score was presented in Table 3. We used the population with a healthy lifestyle score of 0–2 as a reference, and the logistic regression model adjusted for gender, marital status, education, occupation, annual household income, living alone status, major history diseases (hypertension, coronary heart disease, dyslipidemia), systolic blood pressure, and diastolic blood pressure. The likelihood of having a healthy lifestyle score of 5–6 as an adult was higher for experiencing famine in preschool (*p* = 0.018) and school age (*p* = 0.033), with an association effect OR (95% CI) of 1.320 (1.048, 1.663) and 1.300 (1.021, 1.656), respectively. There was no statistically significant association between experiencing famine in infancy and preschool and a healthy lifestyle score of 3–4.

**Table 3:** Associations between different famine exposed stage and healthy lifestyle scores.

**Famine exposed groups**	**Healthy lifestyle scores (3-4)**		**Healthy lifestyle scores (5-6)**	
	**OR (95% CI)**	***P*-value**	**OR (95% CI)**	***P*-value**
Adolescence/adulthood-exposed	1.000		1.000	
Infancy-exposed	1.084 (0.872, 1.348)	0.467	1.014 (0.788, 1.305)	0.912
Preschool-exposed	1.182 (0.964, 1.449)	0.109	1.320 (1.048, 1.663)	0.018
School-exposed	1.406 (1.137, 1.737)	0.002	1.300 (1.021, 1.656)	0.033

Model adjusted for sex, marital status, education, occupation, living alone, major diseases (hypertension, coronary heart disease, dyslipidemia), systolic blood pressure, diastolic blood pressure.

### 3.4 Age balance control

When investigating the relationship between famine exposure and healthy lifestyles in adulthood, the confounding factor of age must be thoroughly considered. Therefore, we excluded participants born before January 1, 1941 (who were adults at the time of famine exposure) from this study to ensure the reliability of our findings. The association between infancy, preschool, and school-age exposure to Chinese famine and the risk of healthy lifestyles compared with age-balanced control group was presented in Supplementary Table S3. Compared with age balanced control group, men exposed to the Chinese famine during infancy periods exhibited a negative association with non-smoking (OR = 0.681, 95%CI: 0.528–0.877) and non-drinking (OR = 0.525, 95%CI: 0.403–0.685). In contrast, women exposed to the Chinese famine during infancy showed a negative association with healthy weight (OR = 0.725, 95%CI: 0.594–0.866) and non-central obesity (OR = 0.808, 95%CI: 0.657–0.992).

## 4 Discussion

In the present study, we examined the association between famine exposure early in life and individual's healthy lifestyle, and further explored gender differences in this association. Male exposure to famine in infancy increased the risk of smoking and alcohol consumption in adulthood, while female exposure to famine in infancy increased the risk of central obesity. In addition, women exposed to famine early in life were more likely to have an unhealthy weight in adulthood. Our study suggested that early life famine experiences were associated with unhealthy lifestyles. Furthermore, findings suggested that infancy exposure to famine was associated with lower healthy lifestyle scores. These findings contributed to a deeper understanding of the complex relationship between famine exposure and healthy lifestyles.

The association of early exposure to the Chinese famine with hypertension, diabetes, cardiovascular disease and metabolic syndrome has been reported (8, 9, 22), but there was still a lack of sufficient evidence on the link between famine exposure and healthy lifestyles. Previous research (23) on Dutch famine has provided some indication that exposure to famine at a young age is associated with unhealthy lifestyle behaviors in later life. This study found that famine early in a woman's life may be associated with a higher prevalence of smoking and physical inactivity later in life, but not with unhealthy diet and alcohol consumption. It is worth noting, however, that the above study was limited in scope, as their study cohort included only female populations experiencing famine and did not explore whether this finding was similar in male populations. It is important to explore gender differences, as men and women respond differently to famine due to different physiological mechanisms.

The results of our study showed that men who experienced famine during infancy were more likely to engage in unhealthy behaviors such as smoking and drinking as adults. During the famine, people faced extreme existential stress and fear, which may have led some to seek out behaviors such as smoking and drinking as a way to relieve stress (24). Social interactions and socializing among men often involved smoking and drinking. Smoking and drinking could be a way for people to bond, reduce isolation and seek social support (25). There was not enough scientific research on the effects of famine on sleep to confirm that men who experience famine were more likely to have more sleep. However, some research suggested that starvation and malnutrition may affect sleep patterns (24). In some animal studies, starvation has been found to be associated with altered sleep patterns. For example, animals may exhibit more sleep behavior when starved, possibly to conserve energy and promote survival (26). However, more research was needed to understand the exact effects of famine on human sleep. In addition, sleep was affected by many factors, including an individual's physical and mental health, as well as environmental conditions (27, 28). Therefore, while there were some studies that imply that famine might affect sleep, the field still needs more research to confirm this relationship and to understand its exact mechanisms.

Our study also found a strong association between famine exposure and healthy physical activity, and showed the same results in age balance control. A study from the Dutch famine found that individuals who experienced malnutrition early in life tended to lead unhealthy lifestyles such as low physical activity in adulthood (29). This was inconsistent with our findings, which might be due to the fact that our respondents were mainly rural dwellers, with occupations mostly in agriculture, and that heavy and strenuous agricultural activities made it possible for the study participants to be more physically active. The effect of famine experience on physical activity needs to be studied further.

The association of famine exposure with unhealthy weight and central obesity was significantly stronger in the women, but this result was not observed in men. A study concluded that experiencing famine early in life was associated with an increased risk of obesity in adulthood in both men and women (30). Our study showed a significantly increased risk of overweight and obesity in women who experienced famine early in life. Similar trends were observed in several studies based on the 1959–1961 Chinese famine (31, 32). A recent meta-analysis showed a significant association between the risk of overweight and obesity in women exposed to malnutrition in early life, but not in men (33). There are several explanations for this gender difference. First, survivor bias and cultural specificity in China could explain why men who survived the famine were more likely than women to be well nourished. Second, some studies suggested that biological differences in sex hormones, body composition, and glucose metabolism have contributed to the famine effect. In addition, the hypothesis of accelerated “growth” or “catch-up growth” after birth suggested that increased growth rates through nutrient-rich diets may lead to overweight or obesity (34). Gluckman's research (35) showed that people who experience famine are more likely to overeat as adults to compensate for previous the lack of experience. One study (36) noted that women who suffered famine had a higher risk of binge eating than men. In women, fat was deposited in the abdomen (37, 38), and intra-abdominal obesity was associated with low testosterone concentrations in men and high androgens in women (39). The study found a higher prevalence of abdominal obesity in both men and women with early exposure to famine (40), which was less consistent with our findings.

Our study indeed has several significant strengths. First, although many studies have investigated the relationship between early-life famine exposure and various diseases in adulthood, few studies have focused on lifestyles, which important contributors to diseases. Second, we sampled an entire county population to explore the association between early-life famine exposure and healthy lifestyles and elucidated gender differences in this relationship with a large sample size. Despite these strengths, our study has several limitations. First, as a cross-sectional study, it could not determine the causal relationship between famine exposure and healthy lifestyle. Second, consistent with other famine studies in China, date of birth was used to define the exposure cohort, but we do not know individual data on the severity of the famine led to its exclusion from our analyses, and Henan Province was the area severely affected during the famine, especially its rural areas. Third, due to the fact that our study was a survey conducted in a rural area and the scarcity of medical equipment resources, we had to resort to self-reporting to obtain lifestyle content, which may have been subject to recall bias.

## 5 Conclusion

In summary, our study found that exposure to Chinese famine early in life was associated with a risk of unhealthy lifestyles. The gender dimension of this effect was characterized by an increased risk of smoking and drinking in adulthood among men who experience famine during infancy, an increased risk of central obesity among women, and a greater likelihood of women experiencing famine early in life and having an unhealthy body weight in adulthood. In addition, experiencing famine after infancy may have higher healthy lifestyle scores. These findings suggested that policymakers should emphasize the strong scientific links between early life conditions and health outcomes in older populations and incorporate them into public health policy considerations, with a focus on nutrition in the early life and women's health.

## Data Availability

The raw data supporting the conclusions of this article will be made available by the authors, without undue reservation.
