# Supplementary material for: The association of famine exposure with healthy lifestyles at different life stages in rural older adults in China
Source: Front Public Health. 2025 Mar 21;13:1533909. doi: 10.3389/fpubh.2025.1533909 (PMC11970580; doi:10.3389/fpubh.2025.1533909)
Supplement: Supplementary file 1 [file Data_Sheet_1.pdf]

The Association of Famine Exposure with Healthy Lifestyles at Different Life Stages in Rural Elderly Population in China

Supplementary Materials:

Table S1. Definitions of healthy and unhealthy lifestyle factors.
Table S2. Characteristics of participants by healthy lifestyle score
Table S3. Age-balanced control regression results
Figure S1. Flowchart on the sample selecting method at each step.

Table S1. Definitions of healthy and unhealthy lifestyle factors.

Lifestyle factors	Healthy lifestyle	Unhealthy lifestyle
**Smoking**	Non-smoking	Smoking
**Drinking**	Non-drinking	drinking
**Physical activity**		
Vigorous exercise	≥75minutes/week	<75 minutes/week
Moderate exercise	≥150minutes/week	<150 minutes/week
**Sleep duration**	6-8 hours	< 6 hours or > 8 hours
**Waist circumference**		
Men	<90	≥90cm
Women	<85	≥85cm
**Body mass index**	18.5-23.9 kg/m^2	< 18.5 kg/m^2 or > 24 kg/m^2

Table S2. Characteristics of participants by healthy lifestyle score

Variables	Total (n=6458)	Healthy lifestyle scores 0-2 （n=943）	3-4 （n=3900）	5-6 （n=1615）	P-value
**Age in survey**	72.0(68.0,76.0)	72(68.0,76.0)	72.0(68.0,76.0)	72.0(68.0,76.0)	< 0.001
**Gender, n (%)**					< 0.001
Male	2303(35.7)	657(69.7)	1211(31.1)	435(26.9)	
Female	4155(64.3)	286(30.3)	2689(68.9)	1180(73.1)	
**Marital status, n (%)**					< 0.001
Married	4817(74.6)	758(80.4)	2903(74.4)	1156(71.6)	
Other	1641(25.4)	185(19.6)	997(25.6)	459(28.4)	
**Annual household income in CNY/years, n (%)**					0.161
≤2840	1616(25.0)	224(23.8)	989(25.3)	403(25.0)	
2841-5000	2127(32.9)	338(35.8)	1243(31.9)	546(33.8)	
5001-10200	1101(17.1)	161(17.1)	655(16.8)	285(17.6)	
≥10201	1614(25.0)	220(23.3)	1013(26.0)	381(23.6)	
**Education level, n (%)**					< 0.001
Illiteracy	2885(44.7)	296(31.4)	1800(46.2)	789(48.9)	
Primary school	2078(32.2)	339(35.9)	1234(31.6)	505(31.3)	
Middle school	1073(16.6)	217(23.0)	626(16.0)	230(14.2)	
High school or above	422(6.5)	91(9.7)	240(6.2)	91(5.6)	
**Occupation, n (%)**					0.045
Agricultural work	4047(62.7)	582(61.7)	2411(61.8)	1054(65.3)	
Nonagricultural work	2411(37.3)	361(38.3)	1489(38.2)	561(34.7)	
**Live alone**					0.001

Table S2.continue.

Variables	Total (n=6458)	Healthy lifestyle scores 0-2 （n=943）	3-4 （n=3900）	5-6 （n=1615）	P-value
Yes	1079(16.7)	131(13.9)	634(16.3)	314(19.4)	
No	5379(83.3)	812(86.1)	3266(83.7)	1301(80.6)	
**Famine exposure groups**					0.017
Infancy-exposed	1215(18.8)	185(19.6)	748(19.2)	282(17.5)	
Preschool-exposed	1487(23.0)	205(21.7)	884(22.7)	398(24.6)	
School age-exposed	1326(20.5)	166(17.6)	841(21.5)	319(19.8)	
Adolescence/Adulthood-exposed	2430(37.7)	387(41.1)	1427(36.6)	616(38.1)	
**Non-smoking**					< 0.001
Yes	5145(79.7)	357(37.9)	3282(84.2)	1506(93.3)	
No	1313(20.3)	586(62.1)	618(15.8)	109(6.7)	
**Non-drinking**					< 0.001
Yes	5743(88.9)	521(55.2)	3622(92.9)	1600(99.1)	
No	715(11.1)	422(44.8)	278(7.1)	15(0.9)	
**Healthy sleep duration**					< 0.001
Yes	3995(61.9)	298(31.6)	2376(60.9)	1321(81.8)	
No	2463(38.1)	645(68.4)	1524(39.1)	294(18.2)	
**Healthy physical activity**					< 0.001
Yes	4446(68.8)	308(32.7)	2726(69.9)	1412(87.4)	
No	2012(31.2)	635(67.3)	1174(30.1)	203(12.6)	
**Normal weight**					< 0.001
Yes	2426(37.6)	60(6.4)	939(24.1)	1427(88.4)	

Table S2.continue.

Variables	Total (n=6458)	Healthy lifestyle scores			P-value
		0-2 （n=943）	3-4 （n=3900）	5-6 （n=1615）	
No	4032(62.4)	883(93.6)	2961(75.9)	188(11.6)	
**Non-central obesity**					< 0.001
Yes	2325(36.0)	78(8.3)	920(23.6)	1327(82.2)	
No	4133(64.0)	865(91.7)	2980(76.4)	288(17.8)	
**Major history diseases, n (%)**					
Hypertension	3282(50.8)	518(54.9)	2041(52.3)	723(44.8)	< 0.001
Coronary heart disease	1073(16.6)	208(22.1)	656(16.8)	209(12.9)	< 0.001
Dyslipidemia	473(7.3)	99(10.5)	359(9.2)	15(0.9)	< 0.001
**SBP, mmHg**	137.5(125.3,150.3)	137.6(125.7,150.0)	138.0 (125.7,150.7)	136.0(124.3,149.0)	< 0.001
**DBP, mmHg**	75.3(68.3,82.3)	76.7(69.5,83.5)	75.7(68.7,82.7)	73.7(66.7,80.4)	< 0.001

Table S3. Age-balanced control regression results

Variables	Total		Men		Women	
	**OR (95% CI)**	**P-value**	**OR (95% CI)**	**P-value**	**OR (95% CI)**	**P-value**
**Non-smoking**						
Age-balanced control	1.000		1.000		1.000	
Infancy-exposed	1.331(1.100,1.611)	0.003	0.681(0.528,0.877)	0.003	0.703(0.306,1.616)	0.407
Preschool-exposed	1.138(0,953,1.359)	0.154	0.825(0.657,1.037)	0.099	0.698(0.313,1.557)	0.380
School-exposed	0.950(0,792,1.140)	0.584	0.848(0.671,1.071)	0.165	0.889(0.368,2.149)	0.794
**Non-drinking**						
Age-balanced control	1.000		1.000		1.000	

Table S3.continue.

Variables	Total		Men		Women	
	OR (95% CI)	P-value	OR (95% CI)	P-value	OR (95% CI)	P-value
Infancy-exposed	0.940(0.749,1.180)	0.596	0.525(0.403,0.685)	< 0.001	0.667(0.276,1.614)	0.369
Preschool-exposed	1.197(0.951,1.507)	0.125	0.909(0.704,1.172)	0.462	1.313(0.483,3.567)	0.594
School-exposed	1.002(0.791,1.269)	0.988	0.936(0.719,1.217)	0.621	0.982(0.366,2.639)	0.972
**Healthy sleep duration**						
Age-balanced control	1.000		1.000		1.000	
Infancy-exposed	1.154(0.990,1.345)	0.066	1.259 (0.969,1.637)	0.085	1.149(0.949,1.392)	0.156
Preschool-exposed	1.103(0.958,1.269)	0.173	1.379(1.085,1.751)	0.009	1.007(0.845,1.201)	0.934
School-exposed	1.020(0.884,1.176)	0.789	1.345(1.053,1.718)	0.018	0.892(0.748,1.065)	0.207
**Healthy physical activity**						
Age-balanced control	1.000		1.000		1.000	
Infancy-exposed	2.180(1.847,2.573)	< 0.001	1.970(1.500,2.588)	< 0.001	2.118(1.712,2.619)	< 0.001
Preschool-exposed	1.894(1.631,2.199)	< 0.001	1.688(1.326,2.147)	< 0.001	1.934(1.594,2.346)	< 0.001
School-exposed	1.725(1.484,2.006)	< 0.001	1.626(1.272,2.077)	< 0.001	1.769(1.458,2.146)	< 0.001
**Normal weight**						
Age-balanced control	1.000		1.000		1.000	
Infancy-exposed	0.813(0.695,0.952)	0.010	1.051(0.808,1.367)	0.713	0.725(0.594,0.886)	0.002
Preschool-exposed	0.857(0.741,0.990)	0.036	1.071(0.844,1.359)	0.572	0.759(0.631,0.913)	0.003
School-exposed	0.886(0.764,1.026)	0.105	1.084(0.850.1.383)	0.514	0.788(0.654,0.949)	0.012
**Non-central obesity**						
Age-balanced control	1.000		1.000		1.000	
Infancy-exposed	0.838(0.716,0.980)	0.027	1.055(0.818,1.360)	0.680	0.808(0.657,0.992)	0.042
Preschool-exposed	0.977(0.846,1.128)	0.750	1.072(0.851,1.351)	0.553	0.965(0.800,1.163)	0.707
School-exposed	0.943(0.814,1.092)	0.433	0.981(0.774,1.243)	0.874	0.915(0.756,1.107)	0.359

Notes: Models were adjusted for marital status, education, occupation, annual household income, living alone status, major diseases (coronary heart disease, dyslipidemia), systolic blood pressure, diastolic blood pressure.

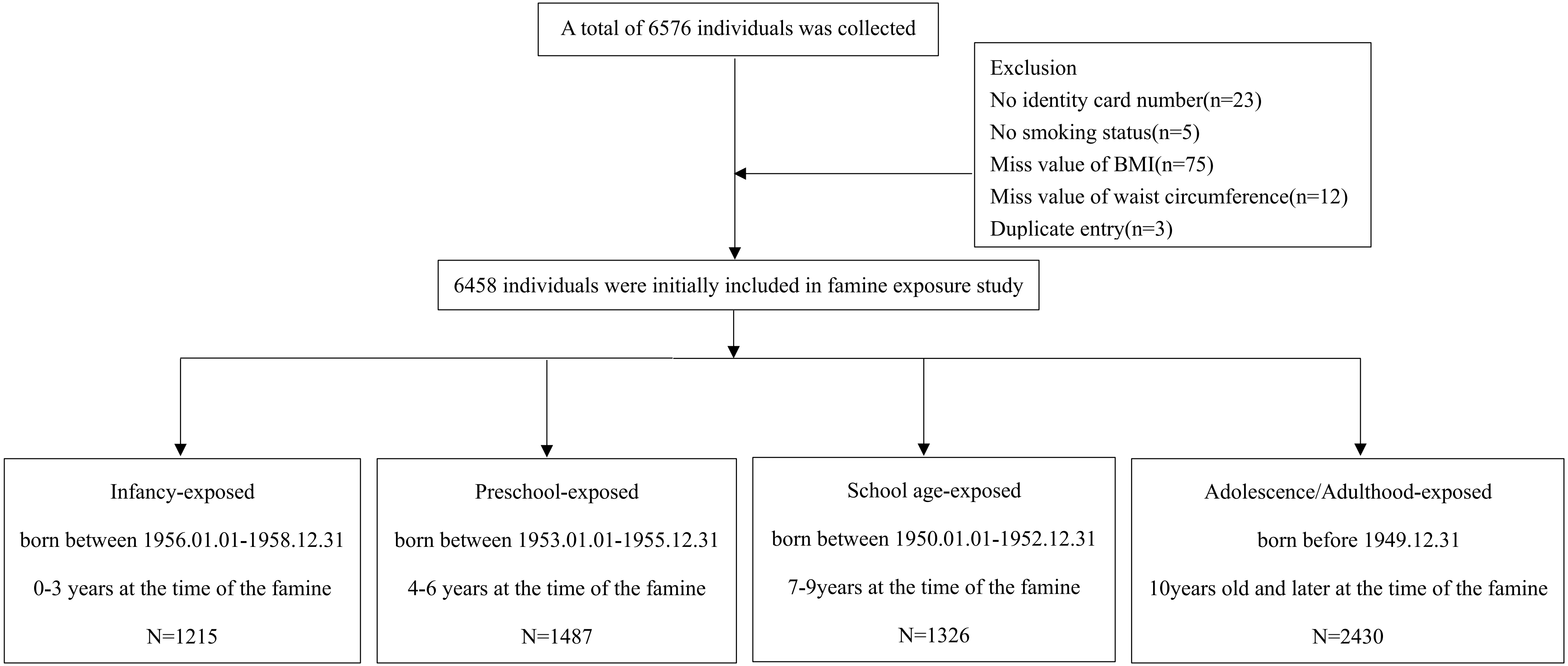

Figure1. Flowchart on the sample selecting method at each step.
